# *Aedes aegypti* breeding site in an underground rainwater reservoir: a warning

**DOI:** 10.11606/S1518-8787.2017051000087

**Published:** 2017-12-04

**Authors:** Patricia Marques Moralejo Bermudi, Fernanda Kowalski, Marcela Mori Menzato, Millene da Cruz Ferreira, Willian Brendo Silva dos Passos, Vivian Janine Ambriola Oku, Aline Kumow, Taís Vargas Freire Martins Lucio, Tamara Nunes Lima-Camara, Paulo Roberto Urbinatti, Francisco Chiaravalloti

**Affiliations:** I Universidade de São Paulo. Faculdade de Saúde Pública. Curso de Graduação em Saúde Pública. São Paulo, SP, Brasil; II Universidade de São Paulo. Faculdade de Saúde Pública. Programa de Pós-Graduação em Saúde Pública. São Paulo, SP, Brasil; III Universidade de São Paulo. Faculdade de Saúde Pública. Departamento de Epidemiología. São Paulo, SP, Brasil

**Keywords:** Aedes, growth & development, Mosquito Vectors, Epidemiological Surveillance, Culicidae, Aedes, crescimento & desenvolvimento, Mosquitos Vetores, Vigilância Epidemiológica, Culicidae

## Abstract

We describe the discovery of *Aedes aegypti* underground breeding site in the Pinheiros neighborhood of São Paulo, SP, during an entomological survey program performed in 2016. Even with intense surveillance and vector control, large numbers of mosquitoes were present in this area. A detailed investigation allowed for the detection of *Ae. aegypti* in an underground reservoir used for rainwater storage. After the implementation of protection screens in the accesses, the presence of the vector was no longer detected. In this study, we discuss the frequent use of this type of reservoir structure and its risk for mosquito production.

## RESUMO

Descreve-se o encontro de criadouro subterrâneo de *Aedes aegypti* no bairro de Pinheiros, São Paulo, SP, ocorrido durante um programa de levantamento entomológico, realizado em 2016. Mesmo com intensa vigilância e controle vetorial, grande quantidade de mosquitos estava presente nessa área. Investigação minuciosa permitiu a detecção de *Ae. aegypti* em reservatório subterrâneo para armazenamento de água pluvial. Após a implantação de telas de proteção nos acessos, não foi mais detectada a presença do vetor. O uso frequente desse tipo de estrutura e o seu risco para a produção de mosquitos é discutido.

## INTRODUCTION

*Aedes (Stegomya) aegypti* (Linnaeus, 1762) originates from the African Continent and is widely distributed in tropical and subtropical areas. It has a holometabolic development, with egg, larva, pupa, and adult phases. Because it is a mosquito highly adapted to the urban environment, its most common breeding sites are artificial containers that accumulate water, such as bottles, tires, cans, and pots^3^. It is considered the main arbovirus vector that causes dengue, chikungunya, zika, and urban yellow fever, whose transmission to humans occurs through the bite of the infected female.

Dengue is caused by a virus with four antigenically distinct serotypes (DENV-1, DENV-2, DENV-3, and DENV-4), and it is a serious public health problem in Brazil, with a high number of suspected and confirmed cases every year. The recent entry of the arboviruses chikungunya (CHIKV) and zika (ZIKV) in the country also made these diseases serious public health problems: the first due mainly to the occurrence, in the chronic phase, of joint impairment and the endurance of symptoms for longer than three months; and the second due to the association with cases of microcephaly and other severe conditions of central nervous system impairment, such as Guillain-Barré syndrome^3^ ^,^ ^5^. In addition, there is considerable concern about the risk of re-urbanization of yellow fever, especially in periods with epizootic events and an increase in the number of human cases of yellow fever.

In the absence of effective vaccines against zika and chikungunya fevers and the existence of a dengue vaccine - the introduction of which needs to be cautious, with a prior assessment of national and local epidemiology^9^ - the best way to control *Ae. aegypti* is by eliminating its breeding sites, thus preventing their proliferation. In this context, we must highlight the role of entomological surveillance in the identification and quantification of the main breeding sites of this vector.

The objective of this study is to report the discovery of an underground breeding site, in 2016, during entomological surveillance activities of *Ae. aegypti* at the Faculdade de Saúde Pública of the Universidade de São Paulo (FSP-USP), located in the western region of the city of São Paulo, state of São Paulo, Brazil.

The FSP-USP has a team responsible for the development of weekly entomological surveillance activities, which include searches for potential and positive breeding sites for *Ae. aegypti,* as well as collections of adult mosquitoes in the inner and outer areas of college buildings. For the identification of breeding sites, lanterns, entomological shells (80 ml for small breeding sites and 350 ml for large breeding sites), and Pasteur pipettes (10 ml) are used to collect larvae and pupae, and plastic pots are used for storage. In the capture of adults, a manual entomological vacuum cleaner, powered by a 12-volt battery, is used.

After collection, both immature and adult forms are referred to FSP-USP's Public Health Laboratory of Entomology, where they are identified in a stereoscopic microscope, according to the dichotomous key proposed by Consoli and Lourenço-de-Oliveira^1^. When possible breeding sites and, in particular, mosquito breeding foci are identified, temporary or permanent measures are adopted to eliminate them, depending on the characteristics of the breeding sites.

In the entomological surveys carried out in March 2016, the cafeteria for the cleaning company's staff was of note, located in the basement of the main building of the FSP-USP, with the aspiration of 64 males and 38 females of *Ae. aegypti,* as well as the identification of underground breeding site, called “reusable water reservoir”, where 158 adults (66 females and 92 males) and one immature *Ae. aegypti* were captured.

This reservoir consists of an underground rainwater storage system and is shown in the Figure, in which we indicate: two lateral access chambers to the reservoir (1 and 3), central access to the reservoir (2); a drain to collect rainwater (4) and a breather (6). The drain inlet and vent are located near the above-mentioned cafeteria's window (5). This illustration was done from photographic records and by means of a 3D drawing, with an isometric view, in the software SolidWorks^®^ 2016.

**Figure:**
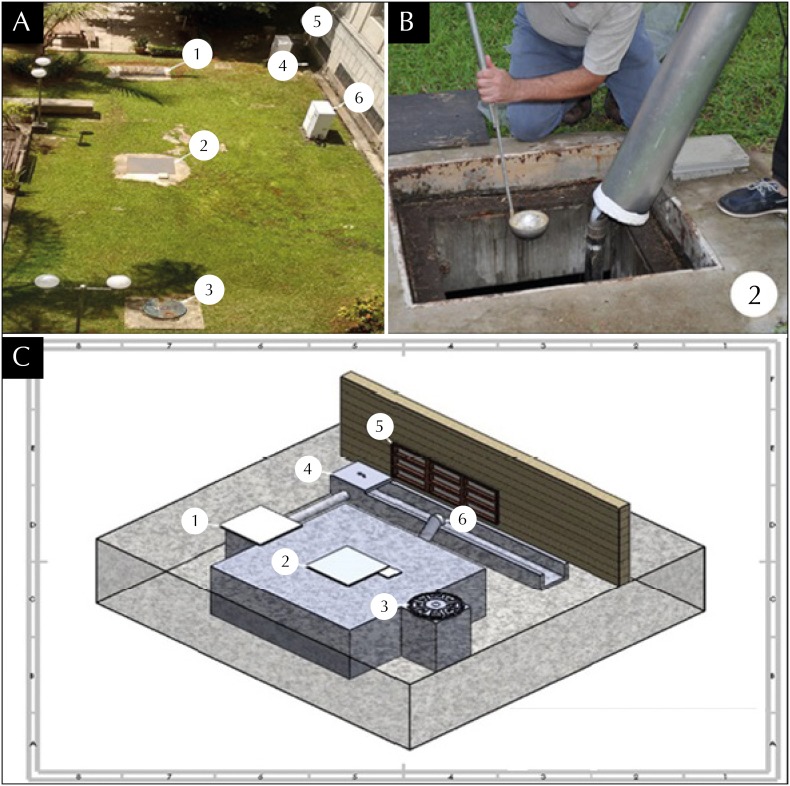
[A] Top view of the underground system, a reservoir of reused water of the Faculdade de Saúde Pública of the Universidade de São Paulo (FSP-USP), *Aedes aegypti* breeding ground where 158 adults were found in March/2016; (1 and 3) lateral access chambers to the reservoir, (2) central access to the reservoir, (4) drain to collect rainwater, (5) window of the FSP-USP underground cafeteria, and (6) reservoir vent. [B] Detail of the central access to the reservoir (2) and of the entomological activities, collection with entomological shell and aspirator, carried out in this place. [C] Isometric view of the underground system. São Paulo, state of São Paulo, 2016.

The first measure adopted to eliminate the subterranean breeding ground and to seal the reservoir entry against mosquitoes was the replacement of the wooden covers (without mosquito fence) for iron covers in the lateral access chamber and in the central access (Figure, items 1 and 2). However, the problem persisted and, in the first survey conducted in April 2016, 78 adults (23 females and 55 males) were captured in the reservoir, indicating the need for a new intervention. As a second measure, protective screens were placed under the cover of the lateral chamber 1 and the central access of the reservoir, resulting in the temporary reduction of collected adult mosquitoes (five females and two males) in the first entomological survey of May 2016. The cover of chamber 3 (Figure) did not require intervention as it provided a complete seal against mosquito entry.

In a new survey, also in May 2016, there were 28 immature forms *of Ae. aegypti* in the reservoir and seven females and four adult males were found in the basement cafeteria. In view of the problem's persistence, a thorough investigation was carried out at the site, confirming the existence of a drainage system for collecting rainwater to supply the reservoir and the vent. Since all other possible access to the reservoir were sealed, it was then inferred that the mosquitoes were using the inlet of the drainage system and the vent (Figure, items 4 and 6) to access and exit the reservoir.

Thus, the third and last intervention to eliminate this breeding ground was adopted, which was the implantation of a system of protection screens at the entrances of the drainage system and the reservoir vent (Figure, items 4 and 6), located near the window of the FSP-USP basement cafeteria (Figure, item 5). In subsequent entomological surveys, carried out in 2016, no more immature forms of the vector were found in the underground reservoir and neither of its adult forms was found in the cafeteria, indicating a resolution of the problem detected in the March survey. However, later surveys continued to detect the presence of immature forms of *Ae. aegypti* in other types of artificial and natural containers, as well as adults in other locations than the underground level of the FSP-USP, showing that the surveillance and control of this vector mosquito should be of a permanent nature.

In the absence of nearby artificial breeding sites, female *Ae. aegypti* can fly large distances in search of places to lay eggs^5^ or even lay eggs in less usual places, such as tree hollows^4^. Additionally, in studies carried out in Australia, it was identified that, in periods of breeding shortage or under unfavorable conditions, female *Ae. aegypti* search underground sites, such as wells, septic tanks, and manholes, to deposit their eggs and seek shelter^2^ ^,^ ^7^.

It should be noted that the identification of the reservoir and its underground connection system as a focus for mosquito breeding was only possible thanks to the systematic performance of entomological surveys in that place, associating the search for breeding sites and the capture of adults. The high number of adults observed, especially males, indicated the presence of a close breeding site, since, in general, the dispersion of males is limited^8^. In addition, this discovery reinforces other findings on the plasticity of *Ae. aegypti* for development in diverse types of breeding grounds, which makes it difficult to control this vector in the country.

The result of this study warns to the potential emergence of unusual breeding sites of *Ae. aegypti* due to solutions for the use of rainwater. These strategies have been adopted in several Brazilian cities and especially in the state of São Paulo, due to the water crisis faced since 2014. One of the ways in which water scarcity has been circumvented is by capturing and storing rainwater in underground cisterns or barrels^6^. As shown in this study, this type of solution can generate *Ae. aegypti* foci and result in an increased risk of occurrence of arboviruses transmitted by this mosquito.
